# Physical Exercise and Gut Microbiota: Implications for Alzheimer’s Disease in Experimental Models: A Systematic Review and Meta-Analysis

**DOI:** 10.3390/jfmk11030287

**Published:** 2026-07-24

**Authors:** María Merino-País, Susana López-Ortiz, Enzo Emanuele, Camillo Imbimbo, Bruno P. Imbimbo, Simone Lista, Alejandro Santos-Lozano

**Affiliations:** 1 i+HeALTH Strategic Research Group, Department of Health Sciences, Miguel de Cervantes European University, 47012 Valladolid, Spain; mmerinop@uemc.es (M.M.-P.); slista@uemc.es (S.L.); 2 2E Science, 27100 Pavia, Italy; enzo.emanuele@2escience.com; 3 Department of Brain and Behavioral Sciences, University of Pavia, 27100 Pavia, Italy; camillo.imbimbo01@universitadipavia.it; 4 Department of Research and Development, Chiesi Farmaceutici, 43122 Parma, Italy; b.imbimbo@chiesi.com; 5 Physical Activity and Health Research Group (PaHerg), Research Institute of the Hospital 12 de Octubre (‘imas12’), 28041 Madrid, Spain

**Keywords:** dementia, mice, treadmill, microbiome, dysbiosis, gut-muscle-brain axis

## Abstract

**Background and Objectives**: The concept of the gut–muscle–brain axis encompasses the intricate, multidirectional interactions between the gut microbiota (GM), physical exercise (PE), and the central nervous system. Within this framework, gut dysbiosis has been implicated in the pathogenesis of Alzheimer’s disease (AD). Given that cognitive functions in AD appear to benefit from PE, it is plausible to hypothesize that these improvements may be partially mediated by PE-induced alterations in GM taxonomy. Therefore, the objective of this study is to evaluate the potential effects of PE in the GM and their implications for AD. **Methods**: A systematic review was conducted in PubMed, Web of Science and Scopus following the PRISMA guidelines up to July 2025 for preclinical controlled trials that assessed the effects of PE on the GM of AD animal models. A random-effects model meta-analysis was performed to estimate the pooled effect of PE on GM frequency or composition. This study received no external funding. **Results**: Eight studies were included in the systematic review (sample size, *n* = 126), of which two could be meta-analyzed. We found that PE significantly reduced Actinobacteria abundance (MD = −0.005%; 95% CI, −0.008 to −0.002; *p* = 0.001) with no statistically significant evidence of heterogeneity (I^2^ = 89.60%, Q = 0.102, *p* = 0.950) or publication bias observed (Begg’s test, *p* = 0.296), but no significant effects were found for other phylums or genera. **Conclusions**: PE appears capable of modulating the GM of animal models with AD in a selective and heterogeneous manner. Further studies are needed to clarify the mechanisms by which this is possible and to determinate its impact on the pathogenesis of the disease.

## 1. Introduction

Alzheimer’s disease (AD) represents the most prevalent form of dementia, with projections indicating a dramatic increase from 50 million cases in 2010 to approximately 113 million by 2050 [1]. This neurodegenerative disorder is distinguished by a progressive decline in cognitive function, which is accompanied by the extracellular accumulation of amyloid-β (Aβ) peptides that aggregate to form senile plaques, and the intracellular hyperphosphorylation of tau proteins, resulting in the formation of neurofibrillary tangles [2,3].

Despite decades of research focused on the amyloid cascade hypothesis, therapeutic approaches targeting Aβ accumulation have shown limited success [4], highlighting the need to explore alternative pathophysiological mechanisms and therapeutic strategies [5]. Notably, growing evidence has highlighted the potential role of gut microbiota (GM) in AD pathogenesis, introducing a new dimension to our understanding of this complex disease [6,7,8]. In this scenario, the concept of the gut–brain axis has emerged as a crucial framework for understanding how alterations in GM composition may influence neurological function and disease progression [9]. Notably, numerous studies have shown that patients with AD exhibit significant alterations in their GM composition, characterized by an increased abundance in pro-inflammatory bacteria and a decrease in beneficial species [10]. Specifically, GM in AD is marked by an increased Firmicutes/Bacteroidetes ratio [11], a reduction in short-chain fatty acid (SCFA)-producing taxa such as *Bifidobacterium* [12] and an enrichment of pro-inflammatory groups like Proteobacteria [13]. The resulting dysbiosis has been linked to increased intestinal permeability, systemic inflammation, and subsequent neuro-inflammation, potentially contributing to AD pathogenesis [14,15]. This link has recently been supported by preclinical analysis in mice [16]. Although an AD mouse model in which the deposition of both beta-amyloid (Aβ) and tau occurs in a similar manner to that observed in sporadic AD patients has not yet been established, these mice play an important role in the development of therapeutic strategies such as modulation of the GM and its effect on the disease [17,18]. In this context, physical exercise (PE) has long been recognized as a powerful non-pharmacological intervention for reducing chronic inflammation and improving overall health [19,20,21,22,23]. Furthermore, its benefits extend to cognitive function, where PE is associated with improved cognitive outcomes across the lifespan and a reduced risk of AD [24,25,26,27]. Accordingly, myokines stimulated by PE, particularly irisin and brain-derived neurotrophic factor (BDNF), have been shown to promote neuroplasticity and cognitive function [28,29,30]. These molecular mediators may in turn interact with the gut microbial ecosystem to influence neural plasticity, anti-inflammatory signaling pathways, and neurogenesis [28].

Within this scenario, research has increasingly revealed a significant connection between PE and GM composition, with the emerging concept of the gut–muscle–brain axis providing a theoretical framework for understanding how PE might influence AD pathophysiology through GM modulation [30,31]. Accordingly, experimental studies in AD transgenic mice have demonstrated that structured PE interventions consistently induce measurable changes in GM composition that correlate with cognitive improvements [32,33,34]. For example, rodent models subjected to voluntary wheel running or forced treadmill exercise show increased abundance of beneficial taxa including *Lactobacillus*, *Bifidobacterium*, and *Akkermansia* species, along with reduced proportions of pro-inflammatory Proteobacteria [35,36]. These PE-induced microbial shifts are associated with enhanced production of neuroprotective metabolites like butyrate, which crosses the blood–brain barrier (BBB) to upregulate BDNF expression in hippocampal regions critical for memory formation [37]. Importantly, the intricate nature of the gut-muscle-brain axis is highlighted by fecal microbiota transplantation experiments, where transfer of PE-modified gut microbes from trained AD mice to sedentary counterparts reproduces cognitive benefits and reduces amyloid burden [38]. However, the precise mechanisms linking PE-induced changes in GM to improved cognitive outcomes in AD remain to be fully elucidated.

Given this background, there is a critical need to systematically evaluate the evidence regarding the effects of PE on GM composition in the context of AD. Current evidence supports the idea that people with AD exhibit dysbiotic patterns that differ from those of their healthy peers and that this dysbiosis triggers systemic inflammation that may accelerate neurodegeneration via the gut–brain axis [39]. Furthermore, in these individuals, PE appears to protect against cognitive decline [40], while also modulating the GM in other populations [41,42]. However, many aspects of this relationship remain unclear. It is not yet well established whether GM dysbiosis is a cause, consequence or bidirectional contributor to AD neuropathology. Also unclear is the extent to which PE-induced cognitive benefits in AD are dependent on GM mechanisms or to which direct PE-induced cognitive benefits in AD are dependent on GM mechanisms or direct neurotrophic and vascular effects of exercise. Moreover, the precise bacterial taxa, metabolites, and signaling pathways that mediate PE-induced neuroprotection in AD are only partially characterized, with considerable variability in findings across different exercise modalities, intensities, and durations. Finally, to date, most mechanistic evidence has been obtained in rodent models and its relevance for human AD remains to be established. Previous systematic reviews have separately evaluated the benefits of PE on clinical AD outcomes [24,43,44] or characterized the general impact of PE on the GM of healthy cohorts [45,46], but none has consolidated how PE modulates the GM specifically within the pathological environment of AD, thereby missing the dual-modulatory effect that this intervention can exert on both systems. Understanding these relationships could provide new insights into the mechanisms underlying the beneficial effects of PE and potentially inform the development of novel therapeutic strategies. We therefore conducted a systematic review and meta-analysis to consolidate current evidence from animal models of AD, with a particular focus on identifying consistent patterns of taxonomic changes and their potential implications for disease pathophysiology.

## 2. Materials and Methods

This work was conducted following the PRISMA recommendations for systematic reviews and meta-analysis (see Supplementary Materials Table S1) [47]. Additionally, review protocol was prospectively registered in the International Prospective Register of Systematic Reviews (registration number PROSPERO: CRD420250639824). All figures were created using the software web Biorender.com.

### 2.1. Databases and Search Strategy

A systematic search was conducted in the PubMed, Web of Science, and Scopus databases, where studies were identified by title and abstract up to 1 July 2025, using the following search syntax: (exercise OR “physical activity” OR training OR “voluntary wheel” OR treadmill) AND (microbiome OR “gut microbiota” OR “gut-brain axis”) AND (Alzheimer OR degenerative OR neurological).

The search was completed with a comprehensive manual search of the reference lists of relevant publications to find additional studies on the subject. The search was conducted independently by two authors (SL-O and MM-P) to avoid any selection bias. The authors independently reviewed the titles, abstracts, and, where applicable, the full texts of the identified publications. After the review, the authors compared their lists; any disagreements were resolved through discussion and agreement among the authors.

### 2.2. Selection Criteria

We included preclinical controlled trials (pCT) that examine the effects of PE on the GM of AD animal models (APP/PS1, Aβ1-40 model and 3xTg-AD). As reported by the participant, intervention, comparison, outcome, and study design (PICOS) process, the inclusion criteria were as follows: (i) participants: animal models for AD (mice or rats), either by genetic variants (transgenic) or drug-induced; (ii) interventions: PE isolated; (iii) comparison group: control group without any intervention such as sedentary animals or sham; (iv) outcome measures: changes in microbiota composition or frequency [including alpha (α) and beta (β) diversity, or relative abundance of specific bacterial taxa at the phylum, family, genus, or species level]; and (v) study design: pCTs conducted in animal models.

The following exclusion criteria were applied: (i) studies written in a language other than English or Spanish, (ii) studies not including mice or rats, and (iii) studies not including an AD model. After removing duplicates, eligibility was finally assessed by (i) reading the title and abstract and (ii) reading the full text if still potentially eligible.

By restricting inclusion to validated AD animal models (APP/PS1 [48], 3xTg-AD [49], and Aβ1-40-induced [50]) this review specifically evaluates PE-induced GM changes within an AD-pathological environment rather than in healthy cohorts, establishing a direct link between GM and AD pathology.

### 2.3. Data Extraction

The following information was collected from each included study: (i) authors’ names and bibliographic references, (ii) year of publication, (iii) randomized protocol, if available, (iv) animals characteristics, (v) number of experimental groups and sample size of each group included, (vi) disease induction method, (vii) housing conditions, (viii) euthanasia method, (ix) PE protocol (i.e., modality, frequency, volume, intensity and duration), (x) voluntary access to the wheel, and (xi) tissue collection, preservation and analysis. When a study included multiple experimental groups, we extracted only the data corresponding to the groups that met the pre-established selection criteria. If critical data were missing or unclear, we attempted to contact the corresponding authors via email. When this was not possible, no assumptions were made, and the missing information was reported as ‘not reported’.

### 2.4. Risk of Bias and Methodological Quality Assessment

Two researchers (MM-P and SL-O) independently assessed the risk of bias of the included studies using the SYstematic Review Center for Laboratory Animal Experimentation (SYRCLE)’s risk of bias (RoB) tool (Cochrane RoB tool adapted for animals) [51]. This tool consists of six domains (selection, performance detection, attrition, reporting bias and other biases) based on empirical evidence and theoretical considerations. The domains are categorized as low, unclear and high risk of bias. For this instrument, the overall bias of each study was classified as “low risk of bias” if all items were classified as “low risk”; “some concern” if at least one area was classified as “some concern”; and “high risk of bias” if at least one area was classified as “high risk” or if several areas were classified as “some concern” and could affect the validity of the results.

To assess the methodological quality, the CAMARADES checklist was used [52]. This tool reports the study quality using ten items (publication in peer-reviewed journal, statement of control of temperature, randomization of treatment or control, allocation concealment, blinded assessment of outcome, avoidance of anesthetics with marked intrinsic properties, use of animals with AD, sample size calculation, statement of compliance with regulatory requirements, and statement regarding possible conflict of interest). Each study was given a quality score out of a possible total of 10 points, and the group median was calculated.

### 2.5. Statistical Analysis

A random-effects model meta-analysis was performed to estimate the pooled effect of PE on GM frequency or composition [53] when the effects of at least three independent experimental comparison groups derived from at least two primary studies were analyzed using the same outcome. The weight assigned to each individual study in the meta-analysis was defined by the standard deviation of the variables and the sample size. To determine the presence of publication bias the Begg’s test was calculated, while I^2^ statistic was used to assess heterogeneity among studies as follows: 0–40% indicated low heterogeneity, 30–60% indicated moderate heterogeneity, 50–90% indicated substantial heterogeneity, and values greater than 75% were considered indicative of considerable heterogeneity [54]. Level of significance was set at *p* < 0.05. All the analyses were performed using MIX 2.0 Pro for Excel software (BiostatXL, Mountain View, CA, USA) [55]. To account for multiple comparisons across the seven outcomes analyzed, a Bonferroni-corrected significance threshold (α = 0.05/7 = 0.007) was additionally applied.

## 3. Results

**Figure 1:**
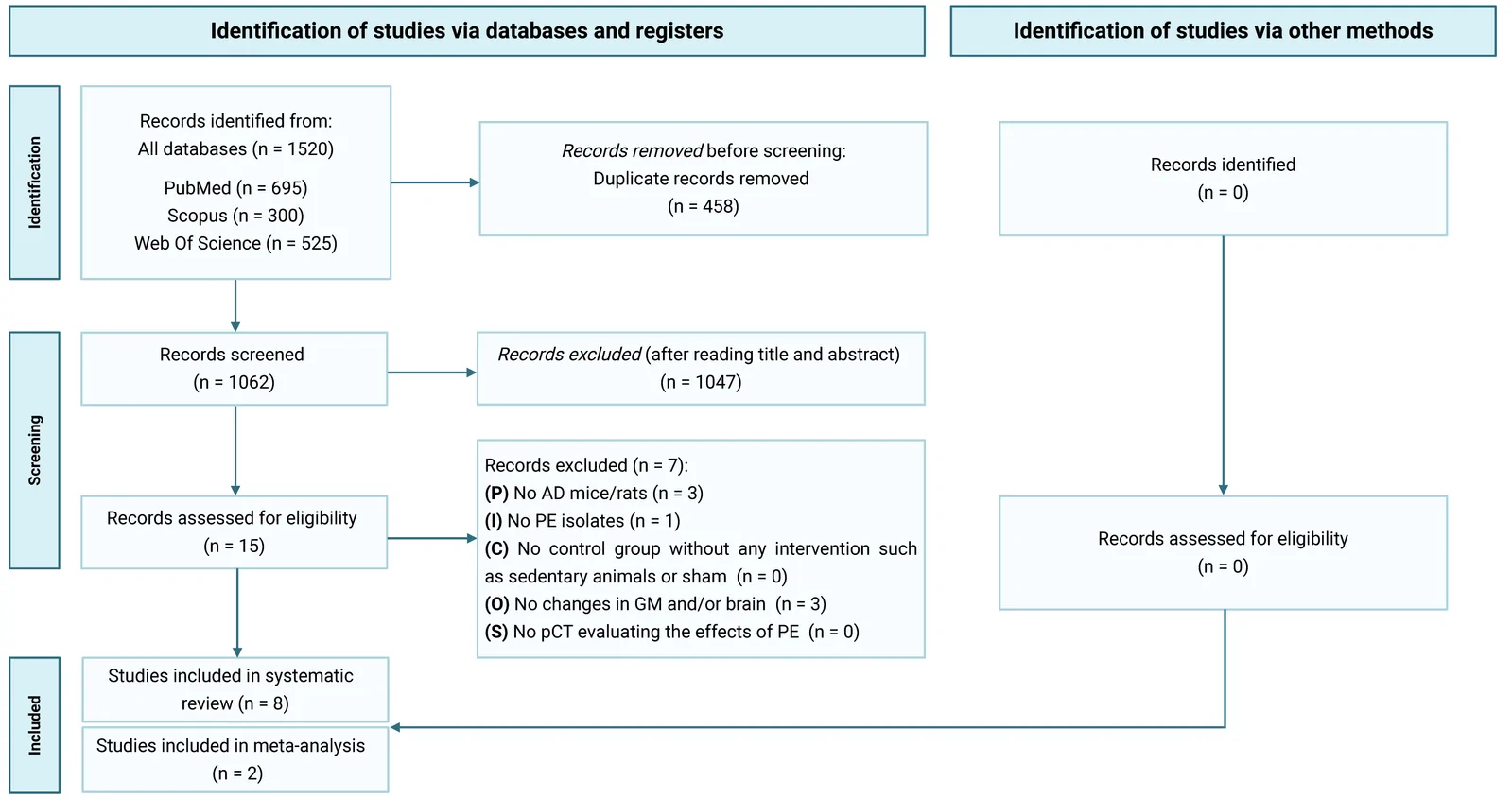
Flowchart of studies included in systematic review and meta-analysis. Created in BioRender. Santos lozano, A. (2026) https://BioRender.com/s4v5393 (accessed on 21 July 2026).

Out of the 1520 retrieved studies, 15 potential articles were identified for inclusion in the systematic review. However, only eight studies were included in the systematic review, of which two could be meta-analyzed, one of them containing two different study populations (see Figure 1).

### 3.1. Studies Characteristics

The main characteristics of the included studies and the detailed protocols of each PE program are summarized alphabetically by the first author’s surname in Table 1. The reported evidence indicates that PE modulates the GM by promoting beneficial taxa and reducing specific pro-inflammatory groups, which consistently aligns with significant reductions in amyloid-β plaque accumulation, lower tau phosphorylation, and increased neuroprotective protein expression in preclinical AD models.

The eight included articles comprise a total sample size of 168 mice, with 84 allocated to an exercise group. All studies used transgenic mouse models of AD. Specifically, six studies used an APP/PS1 model (*n* = 126) [33,56,57,58,59,60], of which two of them used an APP/PS1TG model (*n* = 28) [56,57] and one of them an APPSwe/PS1De9 (*n* = 28) [58]. Other studies used an Aβ1-40 model (*n* = 20) [61] or a 3xTg-AD model (*n* = 22) [32]. In all studies, each group consisted of 6–12 mice ranging in age from 45 days [61] to 24 weeks old [58] and applied treadmill running [32,33,59,61] and interval treadmill running [56,57] with a volume ranging from 30 [32] to 60 min/day [56,57] for four [56,57] or five days a week [32,33,59,61], or voluntary wheel running with open access [58,60]. All training protocols had a duration of four [61], 12 [59,60], 16 [58], 18 [32] or 20 weeks [33,56,57]. Regarding sequencing technology, the microbiome in all included studies was analyzed using 16S rRNA gene sequencing [32,33,56,57,58,59,60,61]. These data highlight a substantial heterogeneity across the compiled literature. Detailed information of the studies included, such as the disease induction and euthanasia method, the housing conditions and the tissue collection, preservation and analysis, can be found in Supplementary Materials Table S2.

#### 3.1.1. Risk of Bias Assessment

The overall risk of bias was high in all pCTs. In all preclinical studies, between four and six items were assessed as high risk of bias. None of the studies included information on allocation concealment or blinding of personnel, nor did they have a previously published or registered protocol (see Appendix A (Table A1)).

#### 3.1.2. Methodological Quality Assessment

The scores on the CAMARADES checklist ranged from 3/10 to 6/10, with an average of 4.625 points. All studies were published in peer-reviewed journals, used animals with AD, and addressed possible conflicts of interest. However, none of the studies reported the sample size calculation, allocation concealment, or the randomization process (see Supplementary Materials Table S3).

**Table 1:** Main characteristics of the included studies.

First Author (Year)	Country	Animal Characteristics	Experimental Groups (*n*)	PE Protocol	Main Results Related to GM	Main Results Related to AD Biomarkers
Abraham et al. (2019) [56]	Hungary	**Sample size**: 32 **Age**: 3 months **Sex**: 100% male **Experimental model**: APP/PS1 transgenic mice (B6C3-Tg (APPswe, PSEN1dE9)85Dbo/Mmjax; APP/PS1TG)	EG (*n* = 8) CG (*n* = 8)	**Modality**: Interval treadmill running **Frequency**: Four times a week **Volume**: Ten cycles of 6 min (60 min/day) **Intensity**: Each cycle consisting of four minutes at high intensity (20 m/min) and two minutes at low intensity (10 m/min) **Duration**: 20 weeks	↓↓↓ Lactobacillus johnsonii and reuteri ↑↑ *Butyrivibrio* *proteoclasticus* and *Roseburia* spp. ↑ Butyrate producing bacteria ↓ Bacteroides fragilis	↓↓ Mean number of plaques in hippocampus and mean % of the areas covered by plaques ↑↑ OGG1 levels and occupied area of OGG1
Jin et al. (2023) [32]	Korea	**Sample size**: 33 **Age**: 3 months **Sex**: 100% male **Experimental model**: 3xTg-AD	EG (*n* = 11) CG (*n* = 11)	**Modality**: Treadmill running **Frequency**: Five times a week **Volume**: 30 min/day **Intensity**: Each session started with a 5 min warm-up at a speed of 5 m/min, increased to 8 m/min for 5 min, and reached a maximum speed of 11 m/min for 20 min. The maximum speed was then increased by 1 m/min every 4 weeks up to a speed of 15 m/min **Duration**: 18 weeks	↑↑↑ Akkermansia muciniphila ↑↑ Bacteroides rodentium ↑↑ Claudin and ZO1 ↓↓ Clostridium amazonense, Alistipes putredinis and Turicibacter sanguinis = Simpson index	↑↑ Time in the target quadrant (MWM testing) ↑↑ ADAM10, hippocampal SOD2 and CAT proteins, Bcl2, hippocampal PSD95, synaptophysin and BDNF ↓↓ Escape latency (MWM testing) ↓↓ Aβ plaque counts in the CA1 and CA3 regions of the hippocampus, APP, BACE1, p-tau205, p-tau231, and total tau in the hippocampus, GFAP-positive cells, TMEM119-positive cells, lower hippocampal caspase 3, caspase 9, and BAX ↑ LRP1 and GLUT1 ↓ IGF1
Rosa et al. (2020) [61]	Brazil	**Sample size**: 40 **Age**: 44–55 days **Sex**: 100% male **Experimental model**: Swiss mice inducted to be Aβ_1–40_ model	EG (*n* = 10) CG (*n* = 10)	**Modality**: Treadmill running **Frequency**: Five times a week **Volume**: 40 min/day **Intensity**: The treadmill speed was progressively increased by 2 m/min/week, starting at 6 m/min at the beginning of the protocol **Duration**: 4 weeks	↑↑↑ Firmicutes ↓↓↓ Bacteroidetes ↓↓↓ Bacteroidetes/Firmicutes ratio	NR
Teglás et al. (2020) [57]	Hungary	**Sample size**: 30 **Age**: 3 months **Sex**: 100% male **Experimental model**: APP/PS1 transgenic mice (B6C3-Tg (APPswe, PSEN1dE9) 85Dbo/Mmjax; APP/PS1	EG (*n* = 6) CG (*n* = 6)	**Modality**: Interval treadmill running **Frequency**: Four times a week **Volume**: Ten cycles of 6 min (60 min/day) **Intensity**: four minutes at 20 m/min and two minutes at 10 m/min **Duration**: 20 weeks	≠≠ GM composition	NR
Wang et al. (2021) [58]	China	**Sample size**: 14 **Age**: 9 weeks **Sex**: 100% male **Experimental model**: APPSwe/PS1De9 (APP/PS1), (B6C3F1 background, APPswe strain)	EG (*n* = 7) CG (*n* = 7)	**Modality**: Voluntary wheel running **Frequency**: Undetermined **Volume**: Undetermined **Intensity**: Undetermined **Duration**: 16 weeks	↑↑ ACE and Chao1 index ↑ *Oscillibacter* ↑ *EU454870_g*, *Oscillibacter*, *EU504554_g*, *EU504066_g*, *EF097061_g*, *EU505046_g* and *EF096172_g* ↓ *Alistipes*	↓↓ GFAP and Ibα1 in cortex ↓↓ Phosphorylation of tau at ser396 and ser404 (p-Tauser396&404) ↑↑ p-GSK3β _at ser9 ↑ ADAM10, synaptophysin and BDNF protein expression and p-AMPK ↓ GFAP and Ibα1 protein expression in hippocampus
		**Sample size**: 14 **Age**: 24 weeks **Sex**: 100% male **Experimental model**: APPSwe/PS1De9 (APP/PS1), (B6C3F1 background, APPswe strain)	EG (*n* = 7) CG (*n* = 7)		↓ *Proteobacteria* and *Tenericutes* ↓ ↓ *Bacteroides* and *Faecalibacterium* ↑ *Allobaculum,* ≠≠ β diversity	↓↓ ADAM10, BACE1 and GFAP ↑ Synaptophysin protein in cortex = p-Tauser396&404, PP2A, p-GSK3β _ser9, CDK5 and p-AMPK
Wei et al. (2025) [33]	China	**Sample size**: 48 **Age**: 3 months **Sex**: Not specified **Experimental model**: APP/PS1	EG (*n* = 12) CG (*n* = 12)	**Modality**: Treadmill running **Frequency**: Five times a week **Volume**: 30 min/day **Intensity**: The protocol started at a speed of 12 m/min for 10 min and progressed to 15 m/min for 20 min. Each session started with a 5 min warm-up at a speed of 5 m/min, increased to 8 m/min for 5 min, and reached a maximum speed of 11 m/min for 20 min. The maximum speed was then increased by 1 m/min every 4 weeks up to a speed of 15 m/min **Duration**: 20 weeks	↑↑ Actinomycota ↓↓ Bacteroidetes = Psychrobacter ↑ Ileibacterium and Faecalibaculum	NR
Yuan et al. (2022) [59]	China	**Sample size**: 48 **Age**: 3 months **Sex**: 100% male **Experimental model**: SPF-grade APP/PS1	EG (*n* = 12) CG (*n* = 12)	**Modality**: Treadmill exercise scheme with gradual increases in exercise intensity **Frequency**: Five times a week **Volume**: 45 min/day **Intensity**: The speed was incrementally increased from 7 to 14 m/min during the first 8 weeks, with a constant speed of 15 m/min for weeks 9–12 with a load intensity of 60% to 73% of the maximum oxygen uptake representing a moderate-intensity aerobic exercise **Duration**: 12 weeks	↑ ACE, Chao, Shannon, and Simpson indices ↑ *Akkermansia* ≠≠ Intestinal microbial composition	↓↓↓ Aβ aggregation ↑↑ Area of hippocampal and cortical niosomes ↓ LPS
Zhang et al. (2023) [60]	China	**Sample size**: 22 **Age**: 10 weeks **Sex**: 100% male **Experimental model**: APP/PS1	EG (*n* = NS) CG (*n* = NS)	**Modality**: Voluntary wheel running **Frequency**: Undetermined **Volume**: Undetermined **Intensity**: Undetermined **Duration**: 12 weeks	↑↑ ACE and Chao1 index ↑↑ *Bacteroidetes*, *Bacteroidales* and *Bifidobacteriales* = Shannon and Simpson index	NR

Notes: ↑/↓: change reported in the text without specifying exact statistical significance; ↑↑/↓↓: statistically significant change (*p* < 0.05); ↑↑↑/↓↓↓: highly significant change (*p* < 0.001); ≠≠ statistically significant difference compared to the control; = no significant differences. Abbreviations: 3xTg-AD, triple-transgenic model of Alzheimer’s disease; _g, genus; Aβ, amyloid-beta; AD, Alzheimer’s disease; ADAM10, A disintegrin and metalloproteinase domain-containing protein 10; APP, amyloid precursor protein; BACE1, beta-site APP cleaving enzyme 1; BAX, Bcl2-associated X protein; BBB, blood–brain barrier; Bcl2, B-cell lymphoma 2; BDNF, brain-derived neurotrophic factor; CA1, cornu ammonis 1; CA3, cornu ammonis 3; CAT, catalase; CDK5, cyclin-dependent kinase 5; CG, control group; EG, experimental group; Ex, exercise; GFAP, glial fibrillary acidic protein; GLUT1, glucose transporter 1; GM, gut microbiota; Ibα1, ionized calcium-binding adapter molecule 1; IGF1, insulin-like growth factor 1; LPS, lipopolysaccharides; LRP1, LDL receptor-related protein 1; m/min, meters per minute; min, minutes; MWM, Morris water maze; *n*, sample size; NR, not reported; NS, not specified; OGG1, 8-oxoguanine glycosylase; *p*, *p*-value; p-AMPK, phosphorylated AMP-activated protein kinase; PE, physical exercise; p-GSK3β _at ser9, glycogen synthase kinase 3 beta phosphorylated at serine 9; PP2A, protein phosphatase 2A; PS1/PSEN1, presenilin 1; PSD95, postsynaptic density protein 95; p-tau, phosphorylated tau; p-Tauser396&404, tau phosphorylated at serine 396 and 404; SOD2, superoxide dismutase 2; SPF, specific pathogen-free; spp., species; TMEM119, transmembrane protein 119; ZO1, zonula occludens 1.

### 3.2. Quantitative Synthesis

**Figure 2:**
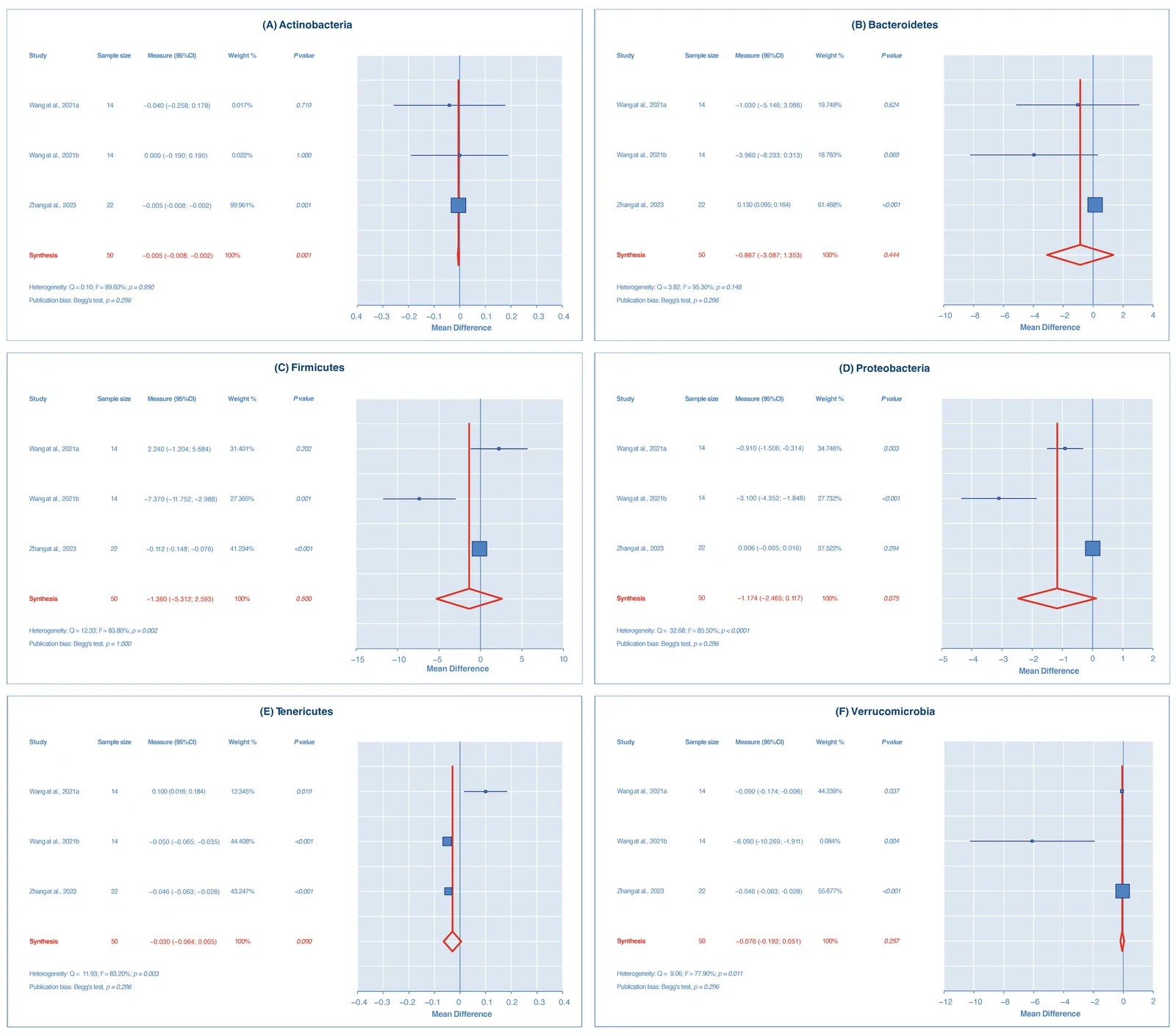
Meta-analysis results for phylums: (**A**) Actinobacteria; (**B**) Bacteroidetes; (**C**) Firmicutes; (**D**) Proteobacteria; (**E**) Tenericutes; (**F**) Verrucomicrobia [58,60].

Two studies [58,60], one with two different subgroup populations [58], investigated the effects of PE on the relative abundance of the phylum Actinobacteria and showed a significant reduction favoring the experimental group [MD = −0.005%, 95% CI: −0.008, −0.002, *p* = 0.001; see Figure 2], with considerable but not statistically significant evidence of heterogeneity (I^2^ = 89.60%, Q = 0.102, *p* = 0.950) and no publication bias (Begg’s test, *p* = 0.296). This result remained statistically significant after applying the Bonferroni-corrected threshold for multiple comparisons (α = 0.007). However, no statistically significant effects were found for the remaining phylums (Bacteroidetes, Firmicutes, Proteobacteria, Verrucomicrobia or Tenericutes), as shown in Figure 2. Significant heterogeneity was found in Firmicutes (I^2^ = 83.80%, *p* = 0.002), Proteobacteria (I^2^ = 85.50%, *p* < 0.001), Tenericutes (I^2^ = 83.20%, *p* = 0.003) or Verrucomicrobia (I^2^ = 77.90%, *p* = 0.011), with no evidence of publication bias.

**Figure 3:**
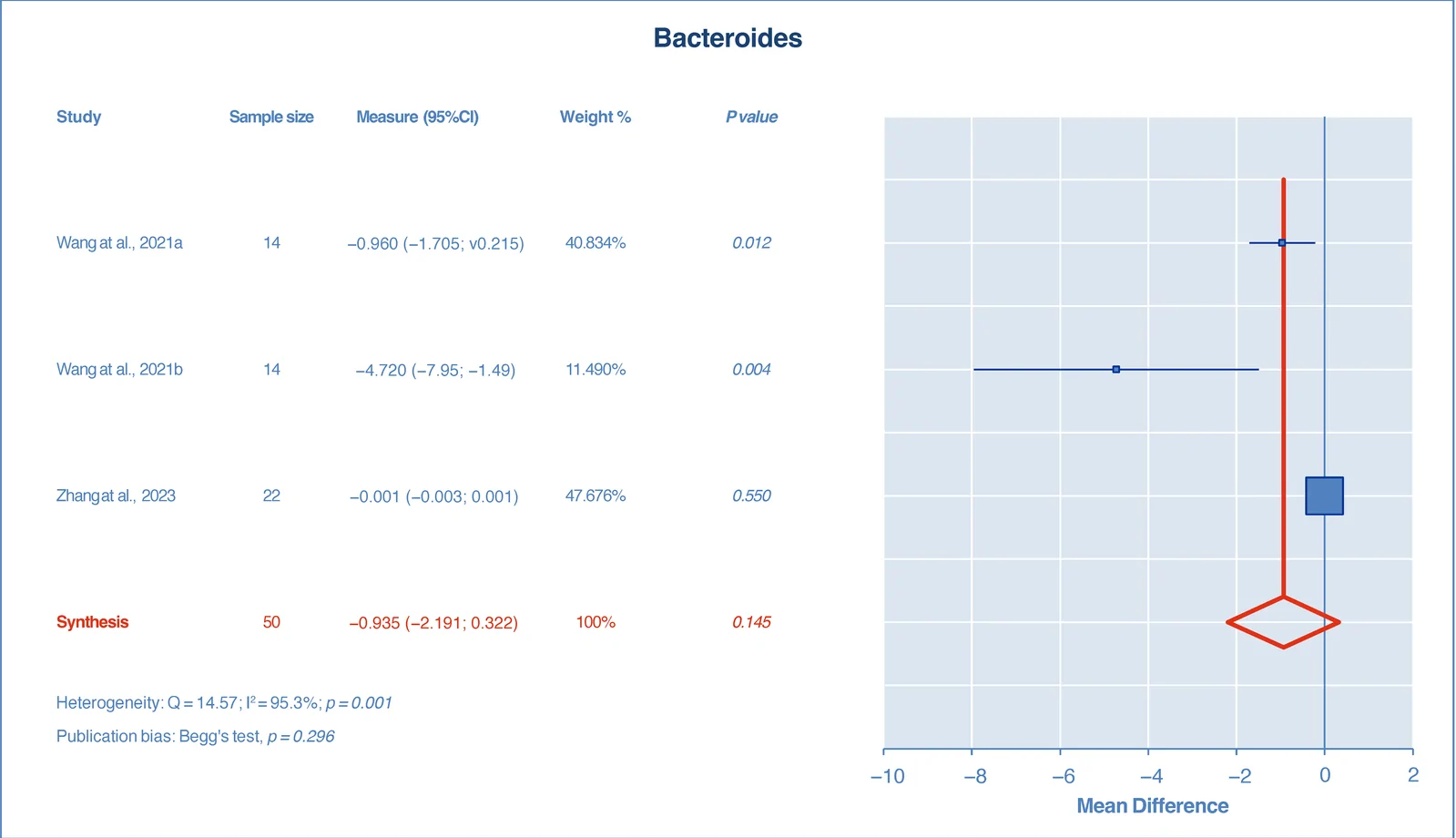
Meta-analysis results for genus Bacteroides [58,60].

No statistically significant differences between groups were found on the relative abundance of the genus Bacteroides (MD = −0.935%, 95% CI: −2.191, 0.322, *p* = 0.145; see Figure 3).

Only two studies reported AD-related biomarkers (e.g., Aβ burden, cognitive performance) that were quantitatively comparable in addition to GM data, therefore the meta-analysis was restricted to GM composition outcomes. Results of AD biomarkers from these studies are summarized narratively in Table 1 and interpreted in relation to observed GM changes in the Discussion Section.

## 4. Discussion

In this systematic review and meta-analysis of pCT, we found that PE significantly reduced Actinobacteria abundance in AD animal models. However, we observed no consistent effects on other major bacterial phyla or genera. Collectively, these findings suggest that PE may exert selective effects on GM composition in AD models. However, these mostly non-significant findings must be interpreted with caution. The lack of studies, the high risk of bias, and the methodological heterogeneity in exercise protocols and sequencing methods are likely to have masked the true effects of PE. This highlights the need for more standardized future studies.

Actinobacteria are Gram-positive, high-G+C-content bacteria that maintain intestinal homeostasis through their capacity to produce bioactive metabolites, including SCFAs and antimicrobial compounds. Members of this phylum, particularly the genus *Bifidobacterium*, are generally considered beneficial commensals that contribute to epithelial integrity and immunomodulation by promoting regulatory T cell differentiation and reducing pro-inflammatory cytokine release [62]. However, in AD, Actinobacteria abundance shows contradictory patterns across studies. Prior research has reported an increased abundance of Actinobacteria in AD animal models and patients, correlating with increased systemic inflammation and metabolic stress. Conversely, other studies demonstrated that specific *Bifidobacterium* species can exert neuroprotective effects through butyrate and γ-aminobutyric acid production, reduction in circulating lipopolysaccharides, and preservation of BBB integrity [15]. The reduction in Actinobacteria observed in our meta-analysis may reflect selective normalization of pro-inflammatory taxa within this phylum, consistent with anti-inflammatory and neuroprotective actions of PE on GM composition and host physiology. However, without genus-level or species-level data, we cannot determine which specific Actinobacteria decreased in response to PE.

Current evidence, predominantly from preclinical research, suggests that GM modulation may influence AD progression [18,63,64] and that regular PE may alter GM composition [35,65]. The literature suggests that PE-induced changes in GM may be associated with immune system regulation [66], reduced inflammation [30,64,67] and improved host metabolism [68], which may impact AD pathology [69]. However, the results of the available studies varied widely, and only two human clinical trials have been reported to date [70,71]. In these studies, PE was combined with diet, stress management and social support, making it difficult to isolate the specific effect of PE on GM.

The mechanisms through which PE confers neuroprotective effects remain incompletely understood, though several pathways have been proposed. Accordingly, PE can induce secretion of exerkines (i.e., signaling molecules released in response to acute and/or chronic PE that exert effects through endocrine, paracrine, and/or autocrine pathways) [72]. Key exerkines include BDNF, irisin, interleukin-6, cathepsin B, myostatin, insulin-like growth factor-1, and the vascular endothelial growth factor. These molecules contribute to cardiovascular, metabolic, immune, and neurological regulation [28], and may also interact with GM dynamics, potentially linking PE with AD-related mechanisms. Once in the bloodstream, exerkines can cross the BBB and preserve neuronal structure and function, improve synaptic plasticity, reduce central inflammation, increase vascularization, and attenuate Aβ deposition [25,69,73,74]. PE also stimulates the secretion of bone-derived factors such as osteocalcin, sclerostin, and osteopontin, which participate in a bone–brain crosstalk that may modulate Aβ pathology and delay AD progression [69].

Early preclinical evidence, such as the study by Matsumoto et al. (2008), showed that voluntary wheel running increased cecal butyrate concentrations in rats, suggesting improved intestinal permeability and reduced inflammation [35,75]. These modifications in cecal microbiota composition may contribute to the beneficial effects of PE on gastrointestinal health [76]. In contrast, sedentary lifestyles are associated with decreased abundance of butyrate-producing bacteria, including *Butyrivibrio proteoclasticus* and *Marvinbryantia formatexigens*, and increased abundance of pro-inflammatory microbes, such as *Clostridium*, *Eubacterium*, and *Roseburia*, which positively correlate with greater Aβ plaques burden in the hippocampus and induce AD pathology in transgenic APP/PS1 mice [56]. Although our meta-analysis did not demonstrate statistically significant changes across most major bacterial phyla, the existing literature suggests that regular PE improves intestinal health by increasing the abundance of beneficial bacteria (e.g., *Firmicutes*, *Lactobacillus reuteri*, *Eubacteria*, *Prevotella*, *Coprococcus*, *Roseburia clostridiales*, *Lachnospiraceae*, *Erysipelotrichaceae*, *Akkermansia muciniphila*) [14,30,31,77,78], reducing intestinal inflammation through reduced circulating LPS [28,30,69,77,78], and strengthening intestinal barrier function [30,69,77,78,79].

However, the relationship between PE and GM deserves further examination, as the potential variability of this interaction requires clarification based on the type and intensity of the PE protocols performed, particularly given the heterogeneous and selective effects observed in our review particularly in relation to the animal models and the main characteristics of the PE interventions.

Mechanisms have been proposed to explain the relationship between PE and GM, particularly through exerkines. In addition, preclinical studies have shown that PE may increase key antioxidant enzymes, such as catalase and glutathione peroxidase, anti-inflammatory cytokines, such as interleukin-10, and anti-apoptotic proteins in intraepithelial lymphocytes, while decreasing pro-inflammatory cytokines (tumor necrosis factor-α and interleukin-17), and pro-apoptotic proteins (caspase-3 and caspase-7). As a result, the overall effect includes reduced intestinal inflammation, increased intestinal blood flow, and modification of bile acid excretion, thereby reducing intestinal dysbiosis [67,72]. Furthermore, PE has been suggested to reduce fecal transit time in the gastrointestinal tract, limiting pathogen contact with the gastrointestinal mucosal layer and, thus, with the circulatory system, thereby decreasing its detrimental effect [65]. Taken together, the available data indicate that PE has the potential to modulate GM by reducing inflammation and dysbiosis, increasing antioxidant enzymes and anti-inflammatory cytokines, and accelerating intestinal transit. Indeed, PE-induced stress, when chronic and moderate, may beneficially modulate GM, whereas excessive stress can lead to harmful allostatic overload [28,80].

Lactate and exerkines are believed to act as key mediators, with lactate serving as a substrate for SCFA-producing bacteria such as *Veillonella* and *Lactobacillus* spp., thereby increasing microbial diversity after PE [28,81]. Previous reviews have identified major inconsistencies across studies, limiting translational potential [82]. High-intensity PE (≥60–70% VO_2_max) may even impair gut integrity by increasing stress hormones and reducing mucosal blood flow [83]. These findings indicate the need to optimize PE protocols to maximize benefits while minimizing potential harm.

PE-induced GM changes may affect different clinical conditions [84]—including cardiovascular [85,86,87], metabolic [88,89], gastrointestinal [90,91] and neurodegenerative diseases, among which AD features predominantly [92,93,94]. However, the composition of the GM is characterized by substantial interindividual variability, modulated by the interplay between host genetic determinants and a wide array of lifestyle and environmental factors, including dietary habits, adiposity, smoking behavior, sleep patterns, pharmacological exposures, and psychological well-being [28,64]. Several factors that modify GM may also influence AD pathogenesis and progression [95,96], suggesting that lifestyle habits affect both AD progression and GM composition, creating bidirectional effects on disease trajectory.

Recent findings demonstrate that GM can modulate immunity, metabolism, and the central nervous system through dynamic bidirectional communication along the gut–brain axis. This axis is a complex communication network that facilitates information exchange between the gut and the brain, involving the central nervous system, the autonomic nervous system, the enteric nervous system, the neuroendocrine system and the immune system, which together maintain the dynamic balance of the intestinal tract, central nervous system, and microbial system [66]. The gut and brain communicate via chemical transmitters, neuronal pathways, and immunoregulatory mechanisms [66,68,97].

Multiple mechanisms connect the gut–brain axis and explain the effect of GM modulation on AD. First, the central nervous system (CNS) can modulate intestinal permeability and motility via the immune pathway, which uses inflammatory cytokines, and the neural pathway, particularly using the vagus nerve, from the brain to the intestine. This nerve is a mixed nerve, containing afferent (80%) and efferent (20%) fibers, allowing bidirectional interaction between brain and intestine. Specifically, vagal afferents detect and transmit metabolites released by GM to the CNS [98]. These pathways can alter GM composition and affect nervous system functions [66,99,100].

Notably, GM can also activate the hypothalamic–pituitary–adrenal axis, triggering cortisol release from the intestine to the brain, regulating microglial activation state [101,102,103] and affecting brain inflammatory state. Separately, gut dysbiosis can promote the production of endotoxins, such as lipopolysaccharide, and inflammatory cytokines and prostaglandins, which directly affect neuro-inflammation. Moreover, GM stimulate the release of various metabolites, such as indole, neurotransmitters (i.e., γ-aminobutyric acid, serotonin, and N-methyl-D-aspartate) and amino acids that enter the bloodstream and reach the brain, where they regulate neuronal function, astrocytes, microglia, and the BBB, allowing GM to alter neuronal circuits through signaling mechanisms and modulate neuronal structure and function through neurotrophic substances. Specifically, when BBB integrity is compromised, several pro-inflammatory cytokines in circulation can enter the central nervous system and induce neuro-inflammation by activating microglia and astrocytes. These alterations can affect the physiological development and function of the brain through elevated inflammatory cytokines such as tumor necrosis factor-α and interleukin-1β [66]. GM imbalances can also be associated with intestinal barrier dysfunction and permeability, which can allow pro-inflammatory cytokines to enter the circulatory system and cross the BBB into the central nervous system, where they can induce neuro-inflammation by activating microglia and astrocytes [66]. Furthermore, reduced intestinal barrier integrity allows pathogen invasion, worsening both intestinal and cerebral inflammatory states, which can lead to Aβ plaque overaccumulation.

Considering the diverse interactions observed—such as the neuroprotective role of PE in AD [104] mediated by exerkine production [28,72,73] and bone-derived factors secretion [69], the influence of regular PE on GM through an increased abundance of beneficial bacteria [30,77,105], reduced intestinal inflammation [64,101,106,107], and improved barrier integrity [14,64,78,107], as well as the multiple bidirectional underlying gut–brain communication [66,68,97]—the hypothesis of a muscle–gut–brain axis appears plausible [66,68,97]. This conceptual framework could explain how PE benefits individuals with AD not only through its direct neurobiological effects on disease progression [108] but also by modulating GM composition and related metabolic functions [25,109,110].

These findings should be interpreted with caution because of inherent limitations. Few preclinical studies met inclusion criteria, restricting our meta-analysis to two studies comprising three experimental subgroups examining the same bacterial species. All included investigations demonstrated high risk of bias and modest methodological quality scores (CAMARADES, 3–6 of 10 points). This risk of bias was due to a partial lack of information on allocation concealment, blinding, randomization, and sample size calculations, which weakens the strength of the available evidence. In addition, the included studies consistently lacked reporting of allocation concealment, blinding procedures, randomization methods, and sample size calculations. Substantial heterogeneity across most phyla (I^2^ = 77–89%) indicates that exercise parameters (type, intensity, volume and duration), AD model characteristics, and housing conditions significantly influence outcomes. Moreover, correction for multiple testing was not consistently applied across the primary studies included, which used different statistical approaches (e.g., ANOVA with post hoc correction compared to uncorrected pairwise comparisons), representing a further source of methodological heterogeneity. Due to the limited number of studies mentioned above, it was not possible to conduct subgroup analyses to clarify which exercise dose might be most effective. Moreover, although we calculated the publication bias in the different meta-analyses, the test conducted may underestimate the bias presence due to the limited number of studies included [111]. Insufficient data precluded subgroup analysis exploring these variables. Furthermore, the absence of species-level taxonomic resolution in most studies prevents identification of specific bacterial taxa modified by exercise. Notwithstanding these caveats, our study offers important strengths. This work represents the first comprehensive systematic review on this topic, consolidating existing knowledge in the scientific literature. Notably, we followed the PRISMA guidelines with prospective registration, ensuring reproducibility and transparency. Given that results of this review derive from animal models, any extrapolation of these findings to patients with AD must be interpreted with caution. Consequently, future research should analyze the effects of specific PE types in humans. Specifically, researchers should conduct adequately powered randomized clinical trials that isolate PE from other lifestyle interventions, employ standardized protocols with defined parameters, and utilize comprehensive microbiome sequencing (shotgun metagenomics and metabolomics rather than 16S rRNA alone).

Longitudinal studies examining dose–response relationships and temporal dynamics of GM changes across exercise modalities are also needed to establish evidence-based exercise prescriptions for AD prevention and management. Investigators should prioritize species-level taxonomic resolution, functional metabolomic profiling, and mechanistic studies determining whether PE-induced GM shifts causally mediate cognitive benefits or represent epiphenomena.

## 5. Conclusions

Our systematic review and meta-analysis suggest that PE can affect GM composition in AD animal models, characterized by a significant reduction in the relative abundance of Actinobacteria but no consistent alterations in other major phyla. These results suggest that the effects of PE on GM may be both selective and heterogeneous. Although the literature suggests that PE may modulate immune regulation, inflammation, and intestinal barrier integrity, further studies are required to elucidate the underlying mechanisms and clarify their translational relevance as a non-pharmacological strategy for AD management. A deeper understanding of the muscle–gut–brain axis may help identify therapeutic targets that integrate PE and GM-based interventions for the management of AD.

## Data Availability

Data will be provided upon reasonable request to the corresponding authors.

## Supplementary Materials

The following supporting information can be downloaded at https://www.mdpi.com/article/10.3390/jfmk11030287/s1, Table S1: PRISMA 2020 checklist; Table S2: Detailed information of the studies included; Table S3: CAMARADES Checklist of the included studies.

## Abbreviations

The following abbreviations are used in this manuscript:

Aβ	Amyloid-β
AD	Alzheimer’s disease
BDNF	Brain-derived neurotrophic factor
BBB	Blood–brain barrier
CNS	Central nervous system
GM	Gut microbiota
pCT	Preclinical controlled trials
PE	Physical exercise
RoB	Risk of bias
SCFA	Short-chain fatty acids
SYRCLE	SYstematic Review Center for Laboratory Animal Experimentation

## Appendix A

**Table A1:** Risk of bias assessment.

	D1	D2	D3	D4	D5	D6	D7	D8	D9	D10	Overall
**Abraham et al. (2019)** [56]	UC	UC	H	H	H	H	H	H	L	UC	H
**Jin et al. (2023)** [32]	UC	UC	H	H	H	H	H	H	L	UC	H
**Rosa et al. (2020)** [61]	UC	UC	H	L	H	H	UC	H	L	UC	H
**Teglás et al. (2020)** [57]	UC	UC	H	UC	H	H	H	H	L	UC	H
**Wang et al. (2021)** [58]	UC	UC	H	UC	H	UC	H	H	L	UC	H
**Wei et al. (2025)** [33]	UC	UC	H	L	H	H	H	H	L	UC	H
**Yuan et al. (2022)** [59]	UC	UC	H	L	H	H	L	H	L	UC	H
**Zhang et al. (2023)** [60]	UC	UC	H	L	H	H	H	H	L	UC	H

Notes: D1. Random sequence generation; D2. baseline characteristics described; D3. allocation concealment; D4. random housing; D5. blinding of participants and personnel; D6. random outcome assessment; D7. blinding of outcome assessment; D8. incomplete outcome data; D9. selective outcome reporting; D10. other source of bias. H, high; L, low; UC, unclear.
