# Supplementary material for: Physical Exercise and Gut Microbiota: Implications for Alzheimer’s Disease in Experimental Models: A Systematic Review and Meta-Analysis
Source: J Funct Morphol Kinesiol. 2026 Jul 24;11(3):287. doi: 10.3390/jfmk11030287 (PMC13510575; doi:10.3390/jfmk11030287)
Supplement: Supplementary file 1 [file jfmk-11-00287-s001.zip › Table S2. Detailed information of the studies included_R1.pdf]

Table S2. Detailed information of the studies included

First author (year)	Disease induction method	Housing conditions	Euthanasia method	Voluntary access to the wheel	Tissue collection, preservation and analysis
Abraham et al., (2019)	NS	NS	Intraperitoneal injection of ketamine (Richter, concentration: 100 mg/ml) /xylazine (Produlab Pharma, concentration: 20 mg/ml) cocktail in a dose of 0.1 ml/10 g bodyweight and transcardially perfused with heparinized ice- cold saline	No; CG was placed on the treadmill and stayed there for five minutes/day on the stationary belt	Fecal samples were collected for microbiome analysis one day before the end of the experiment Microbiome was analyzed by 16S ribosomal rDNA amplicon sequencing and bioinformatics
Teglás et al., (2020)	NS	NS	One dose (0.1 ml/10 g bw) of ketamine via intraperitoneal injection (Richter, 100 mg/ml), and a xylazine (Produlab Pharma, 20 mg/ml) cocktail, and transcardially perfused with heparinized, ice-cold physiological saline	No; animals from the CG, was placed on the treadmill for the same time period as the trained animals, without receiving any PE training	Fecal samples were collected for microbiome analysis one day before the end of the experiment. The samples were stored at – 80°C until processing Microbiome was analyzed by 16S ribosomal rDNA amplicon sequencing and bioinformatics

S2. Cont

First author (year)	Disease induction method	Housing conditions	Euthanasia method	Voluntary access to the wheel	Tissue collection, preservation and analysis
Rosa et al., (2020)	To induce depressive-like behavior, Aβ1-40 was dissolved in sterile PBS (pH 7.4) and aggregated at 37 °C for four days. Mice were lightly anesthetized with 2.5% isoflurane and received a single intracerebroventricular (i.c.v.) injection of either aggregated Aβ1-40 (400 pmol per mouse) or vehicle (PBS) into the left lateral ventricle. The injection was performed perpendicularly through the skull using a 0.4 mm hypodermic needle attached to a Hamilton syringe at the following stereotaxic coordinates from bregma: AP: 0.1 mm, ML: 1 mm, and DV: 3 mm. This procedure was carried out ten days prior to behavioral testing, and mice were allowed a 48-hour recovery period after the i.c.v. injection before commencing the treadmill running protocol.	Animals were housed in groups of 10 animals per plastic cage (41 × 34 × 16 cm) under controlled temperature (21±1 °C) and humidity (50±20 %) with a 12:12 h light/ dark cycle (lights on at 7:00 a.m.)	Mice were anesthetized with pentobarbital (50 mg/kg, i.p.) and transcardially perfused with 0.9 % sodium chloride (NaCl) followed by 4% parafor aldehyde (PFA)	No<br>Treadmill running was scheduled between 1 p.m. and 3 p.m.<br>Mice in the CG was placed on a locked treadmill for the same period of time	Fecal samples were collected from the colon into sterile tubes, which were immediately snap-frozen in liquid nitrogen and subsequently transferred to – 80°C prior to DNA extraction. The fecal sampling was carried out inmediately after the behavioral test. Quantitative real-time PCR assays of 16S ribosomal rRNA gene were performanced

S2. Cont

First author (year)	Disease induction method	Housing conditions	Euthanasia method	Voluntary access to the wheel	Tissue collection, preservation and analysis
Wang et al., (2022)	NS	All mice were raised in standard plastic cages under specific pathogen-free conditions with a 12:12-h light-dark cycle. The temperature was 20–25 .C and humidity was 50–60%. Mice in young EG were housed individually in polypropylegen cages with a running wheel (12-cm diameter) and had free and unlimited access to running wheel. Mice in young CG were housed in a similar polypropylegen cage without the running wheel.	Mice were sacrificed with an overnight fast post the behavioral test	Free and unlimited access to running wheel.	The cecal content were collected and snap-frozen in liquid nitrogen and stored at – 80°C for further 16 S rRNA sequencing and analysis
Yuan et al., (2018)	Not specified	Environment with 12 h of light alternating with darkness and a constant temperature of 22–25 °C	Isoflurane anesthesia was applied the morning after Morris water maze experiment	No	Rectal fecal balls were taken, placed into sterile freezing tubes, immediately immersed into liquid nitrogen, and subsequently stored in a – 80°C freezer until being processed being processed for subsequent 16S rDNA sequencing analysis

S2. Cont

First author (year)	Disease induction method	Housing conditions	Euthanasia method	Voluntary access to the wheel	Tissue collection, preservation and analysis
Zhang et al., (2023)	NS	Animals were housed under controlled conditions (temperature 20–22 .C, food and water ad libitum). Before experiment, mice were acclimated to the condition for one week.<br>For the CG, the mice were fed with modified AIN93-M diet containing 0.07% choline, TMAO group were fed with the above modified AIN93-M diet supplemented with 0.12% TMAO.<br>For the EG, mice were fed with the same diet as the TMAO group and housed individually in polypropylegen cages with a running wheel (12-cm diameter), where mice had free and unlimited access to running wheel.<br>The number of wheel revolution was recorded by an electronic sensor attached to the running wheel, and the mileage was calculated on a weekly base.	The mice were euthanized after overnight fasting	Free and unlimited access to running wheel	Colon tissues and cecal contents were dissected, snap frozen in liquid nitrogen and stored – 80°C.<br>Bacterial DNA was extracted using a PowerMax kit, and the 16S rRNA V3–V4 region was sequenced on the Illumina HiSeq4000 platform.<br>The community composition of each sample and abundance of operation taxonomic units were counted at the levels of kingdom, phylum, class, order, family, genus and species

S2. Cont

First author (year)	Disease induction method	Housing conditions	Euthanasia method	Voluntary access to the wheel	Tissue collection, preservation and analysis
Jin et al., (2023)	Not specified	The animals were housed in a pathogen-free facility at Sungkyunkwan University School of Medicine, with 5 or 6 mice per cage (a constant temperature of 22 °C and a 12:12h light-dark cycle) and free access to standard chow (20% fat, 70% carbohydrates, and 10% protein in kcal, Purina Mills, Seoul, Korea) and tap water	Mice were anesthetized with Alfaxan (80 mg/kg) and Rompun (10 mg/kg) and transcardially perfused with 1 x PBS	No. Mice were prodded by the researcher to make them run if necessary The mice allocated to the CG spent an equal amount of time on the same treadmill without moving	Fecal bacterial DNA was extracted using a PowerMax Soil DNA Isolation Kit and DNA quantity and qualility were checked using a PicoGreen dsDNA Quantitation Kit and Nanodrop. DNA samples were prepared to amplify the V3 and V4 regions (519F-806R) according to the Illumina 16S Metagenomic Sequencing Library protocols
Wei et al., (2025)	Not specified	The mice were housed in pathogen-free conditions with a 12:12h light–dark cycle and had ad libitum access to food and water	Not specified	No<br>The CG were placed in the PE environment but did not undergo training	Genomic DNA was extracted from fecal samples using the MagAtract PowerSoil Pro DAN Kit. Sixteen samples (n = 4 per subgroup) were subjected to microbiota analysis via 16S rRNA gene sequencing

Abbreviations: AD, Alzheimer´s disease; AP, anterioposterior; APP/PS1TG-C, amyloid precursor protein/presenilin 1 transgenic control; Aβ1-40, amyloid-β 1-40; CG, control group; DV, dorsoventral; EG, experimental group; i.c.v., intracerebroventricular; i.p., intraperitoneal; ML, mediolateral; NaCl, sodium chloride; NS, not specified; PBS, phosphate buffered saline; PE, Physical Exercise; PFA, paraformaldehyde; rRNA/rDNA, ribosomal ribonucleic acid/ribosomal deoxyribonucleic acid; TMAO, trimethylamine N – oxide.
