# Supplementary material for: Physical Exercise and Gut Microbiota: Implications for Alzheimer’s Disease in Experimental Models: A Systematic Review and Meta-Analysis
Source: J Funct Morphol Kinesiol. 2026 Jul 24;11(3):287. doi: 10.3390/jfmk11030287 (PMC13510575; doi:10.3390/jfmk11030287)
Supplement: Supplementary file 1 [file jfmk-11-00287-s001.zip › Table S3. CAMARADES Checklist of the included studies_.pdf]

Table S3. CAMARADES Checklist of the included studies.

	Abraham et al., (2019)	Teglás et al., (2020)	Rosa et al., (2020)	Wang et al., (2022)	Yuan et al., (2022)	Zhang et al., (2023)	Jin et al., (2023)	Wei et al., (2025)
**Publication in peer-reviewed journal**	**YES**	**YES**	**YES**	**YES**	**YES**	**YES**	**YES**	**YES**
**Statement of control of temperature**	**NO**	**NO**	**YES**	**YES**	**YES**	**YES**	**YES**	**NO**
**Randomization of treatment or control**	**NM**	**NM**	**NM**	**NM**	**NM**	**NM**	**NM**	**NO**
**Allocation concealment**	**NO**	**NM**	**NM**	**NM**	**NM**	**NM**	**NM**	**NM**
**Blinded assessment of outcome**	**NO**	**NO**	**YES**	**NO**	**NO**	**NO**	**NO**	**NO**
**Avoidance of anesthetics with marked intrinsic properties**	**NO**	**NO**	**NO**	**NM**	**NO**	**NM**	**YES**	**NM**
**Use of animals with AD**	**YES**	**YES**	**YES**	**YES**	**YES**	**YES**	**YES**	**YES**
**Sample size calculation**	**NO**	**NO**	**NO**	**NO**	**NO**	**NO**	**NO**	**NO**

S3. Cont

**Statement of compliance with regulatory requirements**	**YES**	**NM**	**YES**	**YES**	**NM**	**YES**	**YES**	**YES**
**Statement regarding possible conflict of interest**	**YES**	**YES**	**YES**	**YES**	**YES**	**YES**	**YES**	**YES**
**Total (on 10)**	**4**	**3**	**6**	**5**	**4**	**5**	**6**	**4**
